# Perioperative Outcomes of Multivessel Minimally Invasive Coronary Revascularization at a Specialized Center: A Retrospective Cohort of Complete Surgical and Planned Hybrid Strategies

**DOI:** 10.3390/jcm15197424

**Published:** 2026-09-24

**Authors:** Veaceslav Ciobanu, Ihor Krasivskyi, Luca Pizzulli, Eissa Alaj, Marwan Hamiko, Nadejda Monsefi, Farhad Bakhtiary, Kaveh Eghbalzadeh

**Affiliations:** Department of Cardiac Surgery, University Hospital Bonn, 53127 Bonn, Germany

**Keywords:** coronary artery disease, hybrid coronary revascularization, minimally invasive coronary surgery, multivessel coronary disease, MIDCAB, off-pump coronary artery bypass

## Abstract

**Background**: Multivessel minimally invasive coronary revascularization combines surgical myocardial revascularization through a left mini-thoracotomy with, in selected patients, a planned hybrid percutaneous component. Evidence from contemporary single-center cohorts remains limited. **Methods**: We retrospectively analyzed 180 Heart Team-selected patients treated with a multivessel minimally invasive strategy at the Department of Cardiac Surgery, University Hospital Bonn, between December 2021 and February 2025. The cohort comprised complete surgical revascularization and planned hybrid strategies. **Results**: Complete surgical revascularization was documented in 121/180 patients (67.2%), while 59/180 (32.8%) underwent planned hybrid revascularization. No in-hospital deaths occurred (0.0%; exact 95% CI, 0.0–2.0%). Stroke occurred in 2/180 (1.1%), re-thoracotomy for bleeding in 3/180 (1.7%), wound infection in 3/180 (1.7%), and AKI in 8/180 (4.4%). Three patients received postoperative dialysis; two were dialysis-dependent before surgery and one required new postoperative renal replacement therapy (0.6%). Postoperative atrial fibrillation was documented in 7 patients (3.9%). Post-discharge follow-up information was available for 140/180 patients (77.8%), with three recorded deaths. **Conclusions**: This cohort demonstrates that a Heart Team-driven multivessel minimally invasive coronary revascularization program incorporating both complete surgical and planned hybrid strategies can be implemented with favorable observed perioperative outcomes in carefully selected patients. At the same time, the results should not be interpreted as evidence that MV-MIDCAB or hybrid coronary revascularization is superior to conventional CABG.

## 1. Introduction

Coronary artery bypass grafting (CABG) remains an established revascularization strategy for selected patients with multivessel or left main coronary artery disease. Contemporary guidelines emphasize individualized selection of CABG, percutaneous coronary intervention (PCI), or combined approaches according to clinical status, coronary anatomy, anticipated completeness of revascularization, surgical risk, and patient preferences, with multidisciplinary Heart Team decision-making when the optimal strategy is uncertain [1,2].

Minimally invasive direct coronary artery bypass (MIDCAB) avoids median sternotomy and cardiopulmonary bypass while preserving the established role of internal mammary artery grafting. Multivessel minimally invasive coronary revascularization extends this concept to additional coronary targets through a left mini-thoracotomy and may be combined with PCI as planned hybrid coronary revascularization (HCR) [3,4]. HCR remains a specialized strategy with limited randomized evidence and substantial variation in patient selection and procedural sequencing [4].

The present study was undertaken to describe contemporary perioperative outcomes during implementation of a multivessel minimally invasive coronary revascularization program at a specialized tertiary cardiac surgical center. The primary objective was to characterize observed perioperative outcomes, technical implementation, completeness of surgical revascularization, and the use of planned hybrid treatment.

## 2. Materials and Methods

### 2.1. Study Design

This retrospective single-center cohort included 180 patients treated with a Heart Team-selected multivessel minimally invasive coronary revascularization strategy at the Department of Cardiac Surgery, University Hospital Bonn, Germany, between December 2021 and February 2025.

### 2.2. Patient Selection and Heart Team Pathway

Patients entered the pathway after referral for coronary revascularization and preliminary consideration of a minimally invasive multivessel approach. All candidates were discussed by a multidisciplinary Heart Team consisting of cardiac surgeons and interventional cardiologists. Selection favored patients in whom the left coronary circulation could be accessed and grafted through a left mini-thoracotomy, additional target vessels were technically accessible, and the planned surgical or hybrid strategy was considered capable of achieving the intended myocardial revascularization without an anticipated median sternotomy.

The Heart Team considered coronary distribution and target-vessel accessibility, left main disease, ventricular function, clinical presentation, urgency, diabetes and other major comorbidities, surgical risk, previous PCI, anticipated completeness of revascularization, chest-wall and pulmonary factors, and patient-specific considerations.

Patients were excluded if they were hemodynamically unstable, required emergency salvage surgery, had previous cardiac surgery, had prior thoracic radiation, or had significant left subclavian artery stenosis precluding safe use of the internal mammary artery. The EuroSCORE II urgency definitions were used as the conceptual framework: elective surgery was planned admission; urgent surgery required treatment during the current admission; emergency surgery was required before the beginning of the next working day; salvage surgery involved pre-induction cardiopulmonary resuscitation [5].

### 2.3. Operational Definition of Multivessel Minimally Invasive Revascularization

For this study, multivessel minimally invasive coronary revascularization was defined as surgical revascularization through a left mini-thoracotomy with at least two distal coronary anastomoses, without a planned median sternotomy. Surgical targets could include the LAD, diagonal branches, circumflex/obtuse marginal branches, and right coronary/PDA/posterolateral targets. The number of distal anastomoses was recorded separately from the conduit configuration. Sequential anastomoses and composite T-grafts were permitted.

Two treatment pathways were prespecified clinically: (1) complete surgical revascularization, in which all targets designated for surgical treatment by the Heart Team were grafted through the minimally invasive approach; and (2) planned hybrid revascularization, in which the surgically accessible left coronary circulation was treated surgically and selected remaining lesions, commonly in the right coronary distribution, were intended for PCI. PCI-treated vessels were not counted as surgically revascularized. The completeness variable was derived retrospectively from the operative documentation and Heart Team treatment plan.

### 2.4. Planned Hybrid Coronary Revascularization

Planned hybrid treatment was selected when complete surgical treatment through the mini-thoracotomy was considered technically challenging or clinically unnecessary. The surgical component was performed first or, when clinically indicated, in the context of preceding PCI. The institutional protocol permitted aspirin and clopidogrel to be continued for urgent surgery when clinically required, whereas elective surgery after PCI was generally deferred for approximately six weeks when feasible to permit discontinuation of dual antiplatelet therapy. Patient-level timing, lesion-level PCI anatomy, and P2Y12 final-dose timing were not systematically retained in the archived surgical database; these variables are therefore reported as unavailable rather than reconstructed from incomplete records.

### 2.5. Surgical Technique and Technical Implementation

The operative technique has been described previously [6,7,8]. Briefly, the procedure was performed through a left mini-thoracotomy with off-pump coronary anastomoses. Internal mammary arteries were harvested conventionally or thoracoscopically. Composite T-grafts using the right internal mammary artery, radial artery, or saphenous vein were used according to the planned revascularization strategy. The aortic no-touch principle was used whenever feasible.

Technical success was defined as completion of the planned surgical anastomoses through the intended minimally invasive access without unplanned conversion to cardiopulmonary bypass.

### 2.6. Ethics

The study was conducted in accordance with the Declaration of Helsinki. Institutional Review Board Statement: Ethical review and approval were waived for this study due to its retrospective nature. According to § 15(1) of the Professional Code for Physicians in North Rhine and § 6(2) sentence 1 of the North Rhine–Westphalia State Data Protection Act (GDSG NRW), formal ethics approval is not required for retrospective studies of this type.

### 2.7. Data Collection and Outcomes

Clinical, perioperative, and postoperative variables were extracted from the institutional electronic medical record and the prospectively maintained cardiac surgery database. The primary endpoint in the original manuscript was all-cause in-hospital mortality. Secondary outcomes included stroke, re-thoracotomy for bleeding, wound infection, acute kidney injury (AKI), postoperative renal replacement therapy, postoperative atrial fibrillation, delirium, extracorporeal membrane oxygenation (ECMO), mechanical ventilation, intensive care unit (ICU) stay, and hospital stay.

AKI was defined according to Kidney Disease: Improving Global Outcomes (KDIGO) criteria. Stroke was defined as a new focal neurological deficit lasting >24 h and confirmed by neurological assessment and cerebral imaging. Wound infection included superficial or deep surgical-site infection requiring antibiotic therapy and/or surgical treatment. Delirium was based on routine clinical recognition by ICU and cardiac surgical teams. Postoperative atrial fibrillation was identified from the postoperative rhythm record.

### 2.8. Statistical Analysis

The analysis was descriptive and performed using IBM SPSS Statistics (version 28.1) and an independent data-audit workflow. Continuous variables were summarized as median and interquartile range (IQR) because several perioperative variables were skewed. Categorical variables were reported as counts and percentages. Exact binomial 95% confidence intervals (Clopper–Pearson) were calculated for binary outcomes, including zero-event outcomes. No propensity-score analysis was performed because the cohort contained no contemporaneous alternative-treatment group; propensity matching within a single treatment arm would not address confounding by treatment choice. All analyses were conducted using IBM SPSS Statistics, version 28.1 (SPSS Inc., Chicago, IL, USA).

## 3. Results

### 3.1. Study Population and Baseline Characteristics

Of 236 patients (Figure 1) screened within the minimally invasive multivessel pathway, 56 were excluded and 180 proceeded to Heart Team-selected MV-MIDCAB (Table 1). The archived screening record did not retain more granular reasons for the 21 patients categorized as other/not meeting criteria; the revised flow diagram makes this limitation explicit. The cohort had a median age of 68 years, median BMI of 27.0 kg/m^2^, and median EuroSCORE II of 1.24%. Left main disease was present in 52 patients (28.9%), previous PCI in 47 (26.1%), diabetes in 52 (28.9%), NSTEMI in 38 (21.1%), and STEMI in 7 (3.9%). The cohort was therefore predominantly at low predicted operative risk despite including urgent and acute coronary syndrome presentations.

### 3.2. Operative Strategy and Completeness of Revascularization

Complete surgical revascularization was reported in 121/180 patients (67.2%); 59/180 (32.8%) underwent a planned hybrid strategy (Table 2). Two distal anastomoses were performed in 114 patients (63.3%), three in 63 (35.0%), and four in 3 (1.7%). The aortic no-touch technique was used in 179 patients (99.4%). One patient required conversion to cardiopulmonary bypass (0.6%). Bilateral internal mammary artery grafting was used in 42 patients (23.3%), total arterial revascularization was documented in 84 (46.7%), T-graft configuration in 151 (83.9%), endoscopic vein harvesting in 30 (16.7%), and thoracoscopic IMA harvesting in 83 (46.1%).

### 3.3. Coronary Targets and Hybrid Strategy

In the operative-note audit of 179 classified records, LAD targets were documented in 177 cases, diagonal targets in 60, circumflex/obtuse marginal targets in 120, and right coronary/PDA/posterolateral targets in 62. The 59 planned hybrid patients were selected by the Heart Team when complete surgical treatment was considered technically challenging or clinically unnecessary. One planned-hybrid patient had incomplete data for subgroup comparison and was therefore excluded from Table 3.

### 3.4. Early Postoperative Outcomes

No in-hospital deaths occurred (0/180; exact 95% CI, 0.0–2.0%), Figure 2. Stroke occurred in 2/180 (1.1%; 95% CI, 0.1–4.0%), re-thoracotomy for bleeding in 3/180 (1.7%; 95% CI, 0.3–4.8%), wound infection in 3/180 (1.7%; 95% CI, 0.3–4.8%), and AKI in 8/180 (4.4%; 95% CI, 1.9–8.6%), Table 4. Three patients received postoperative dialysis; two were already dialysis-dependent before surgery and one required new postoperative renal replacement therapy. Thus, new dialysis-requiring AKI occurred in 1/180 (0.6%; 95% CI, 0.0–3.1%).

Postoperative delirium was clinically recognized in 24/180 patients (13.3%; 95% CI, 8.7–19.2%). Postoperative atrial fibrillation was documented in 7/180 patients (3.9%; 95% CI, 1.6–7.8%); among 179 records with paired preoperative and postoperative rhythm data, four patients developed atrial fibrillation from preoperative sinus rhythm. One patient required temporary VA-ECMO support and was successfully weaned. Two episodes of advanced life support with return of spontaneous circulation were recorded. One of these occurred in the same patient who also had the only documented conversion to CPB, re-thoracotomy for bleeding, low cardiac output, and ECMO support. The archived structured dataset did not contain sufficient timing and causal detail to adjudicate the precipitating mechanism or neurological outcome of these events.

Operative time was median 192 (175–218) min; ICU stay was median 1 (1–2) days and hospital stay 4 (4–6) days. Vasoactive support at ICU handover was documented in approximately 84% of patients.

### 3.5. Follow-Up

Post-discharge follow-up information was available for 140/180 patients (77.8%), and three deaths were recorded among these patients.

## 4. Discussion

The present study describes the contemporary implementation of a multivessel minimally invasive coronary revascularization program in a Heart Team-selected cohort of 180 patients treated at a specialized tertiary cardiac surgery center. The most relevant finding is that a minimally invasive multivessel strategy could be performed with a low observed rate of major perioperative complications despite the inclusion of multivessel disease and selected patients with left main disease and previous PCI. Importantly, however, the cohort represents a treatment pathway rather than a homogeneous surgical procedure: 121 patients (67.2%) underwent complete surgical revascularization, whereas 59 patients (32.8%) were treated according to a planned hybrid strategy combining minimally invasive surgery with subsequent or preceding PCI. Consequently, the observed outcomes should be interpreted as results of an integrated minimally invasive revascularization program rather than as evidence for the superiority of MV-MIDCAB over conventional CABG or PCI [10].

The patient selection process is central to the interpretation of these findings. Contemporary guidelines emphasize individualized revascularization decisions based on coronary anatomy, clinical presentation, operative risk, feasibility of complete revascularization, and multidisciplinary Heart Team assessment [1,2,11]. The present cohort reflects this principle. Patients were specifically selected when the coronary anatomy and target-vessel accessibility were considered suitable for a left mini-thoracotomy approach, while patients with hemodynamic instability, previous cardiac surgery, prior thoracic radiation, or significant left subclavian disease were excluded. Despite the inclusion of patients with left main disease, NSTEMI, STEMI, and urgent presentations, the median EuroSCORE II was only 1.24%. Thus, the favorable perioperative results should be considered within the context of a carefully selected population with predominantly low predicted operative risk. Similar selection and institutional expertise have been recognized as important determinants of outcomes in minimally invasive coronary surgery [10,11,12].

A particularly notable finding was the absence of in-hospital mortality. Although this represents an encouraging observation, a zero-event rate in a cohort of 180 patients should not be interpreted as proof of procedural safety or superiority. Likewise, the observed stroke rate of 1.1% and re-thoracotomy rate of 1.7% indicate that clinically relevant complications remain possible even within a highly selected minimally invasive pathway. The almost universal use of the aortic no-touch technique (99.4%) is of particular interest. Avoidance of aortic manipulation has been associated with a reduction in perioperative neurological events in comparative analyses of CABG strategies [3,13]. In the present cohort, however, the absence of a comparator prevents attribution of the observed stroke rate specifically to the no-touch strategy. Nevertheless, the technical feasibility of maintaining an essentially aortic no-touch approach in a multivessel minimally invasive program represents an important characteristic of the operative concept.

The renal and neurological outcomes also warrant consideration. AKI occurred in 8 patients (4.4%), while only one patient (0.6%) required newly initiated postoperative renal replacement therapy. Two of the three patients receiving postoperative dialysis were already dialysis-dependent before surgery and therefore should not be classified as having new dialysis-requiring postoperative AKI. This distinction is clinically relevant and avoids overestimation of surgery-associated renal morbidity. 

The operative data further demonstrate that the program was not limited to conventional single-vessel MIDCAB. Two distal anastomoses were performed in 63.3% of patients, three in 35.0%, and four in 1.7%. T-graft configurations were used in 83.9% of patients, while total arterial revascularization was achieved in 46.7% and bilateral IMA grafting in 23.3%. These findings illustrate the technical flexibility required to extend minimally invasive surgery beyond isolated LIMA-to-LAD grafting. Previous institutional experience has demonstrated the feasibility of MIDCAB through a left mini-thoracotomy, including increasingly complex multivessel strategies and alternative conduit configurations [6,7,14]. Earlier multicenter and long-term observational studies have likewise suggested that minimally invasive coronary surgery can provide durable clinical outcomes when performed in experienced programs [10,11,12,15,16]. Nevertheless, the present study was not designed to assess graft patency, and the available intraoperative flow measurements cannot substitute for systematic angiographic follow-up.

The planned hybrid strategy represents another important aspect of this cohort. Nearly one-third of patients were intentionally treated with a hybrid approach because complete surgical treatment through the minimally invasive access was considered technically challenging or clinically unnecessary. Hybrid coronary revascularization is increasingly recognized as an individualized Heart Team strategy that combines the advantages of surgical revascularization of selected coronary territories with PCI for anatomically suitable remaining lesions [4,14,17,18]. The rationale is particularly relevant when the LAD or other surgically accessible territories can be treated effectively with arterial grafting, while lesions in other coronary distributions may be more appropriately addressed percutaneously. The contemporary literature suggests that HCR can achieve clinical outcomes comparable to conventional CABG in selected populations, but the evidence remains heterogeneous and is influenced substantially by patient selection, lesion anatomy, procedural sequencing, and antiplatelet management [14,15,17,19,20,21].

The descriptive differences between the complete-surgical and planned-hybrid pathways also illustrate why direct subgroup comparisons would be potentially misleading. Patients undergoing planned hybrid treatment had fewer distal surgical anastomoses and a longer median operative time, while the hybrid group showed numerically higher rates of AKI and postoperative atrial fibrillation. These observations should not be interpreted as treatment effects because treatment allocation was determined by the Heart Team according to anatomical and clinical considerations. In particular, the decision to use PCI for selected residual lesions inherently identifies a different anatomical and procedural population. This is consistent with the broader literature emphasizing the importance of appropriate patient selection and multidisciplinary decision-making in HCR [13,22].

The high rate of aortic no-touch surgery and the frequent use of composite grafting may also have practical implications for specialized minimally invasive programs. Composite T-grafts allow multiple coronary territories to be addressed without requiring proximal aortic anastomoses and therefore complement the no-touch concept. At the same time, these techniques increase technical complexity and place greater demands on conduit planning, graft length, target-vessel exposure, and intraoperative graft assessment [22]. 

The findings should also be interpreted in the context of programmatic expertise. Minimally invasive multivessel coronary surgery has a recognized learning curve, and previous reports have demonstrated that outcomes are closely related to institutional and surgeon experience [10,11,16]. The present study was conducted in a specialized cardiac surgery center with an established minimally invasive coronary program, and almost half of the procedures incorporated thoracoscopic IMA harvesting. The current dataset, however, did not contain a formal surgeon-specific learning-curve analysis or sufficiently detailed annual institutional volume data. Therefore, the results should not be generalized directly to centers without comparable infrastructure, expertise, or a coordinated cardiac surgery–interventional cardiology pathway.

From a clinical perspective, the present results support the concept that multivessel minimally invasive coronary revascularization can be incorporated into a contemporary Heart Team-based treatment algorithm for carefully selected patients. The observed combination of low in-hospital mortality, low stroke incidence, low conversion rate, short median ICU and hospital stays, and the feasibility of both complete surgical and hybrid strategies is encouraging. Nevertheless, the appropriate interpretation is one of feasibility and observed perioperative performance, rather than comparative effectiveness. Current ESC and ACC/AHA/SCAI recommendations support individualized selection among CABG, PCI, and combined approaches according to anatomy, clinical characteristics, operative risk, and expected completeness of revascularization [1,2,18].

## 5. Limitations

Several limitations deserve particular emphasis. The retrospective single-center design introduces selection and information bias and limits external validity. The screening population itself consisted of patients considered for a minimally invasive pathway and therefore does not represent the complete population of patients undergoing CABG at the institution. Standardized SYNTAX scores and detailed lesion-level anatomical data were unavailable, and the exact timing and anatomical details of PCI were not systematically retained. Several clinically relevant endpoints, including perioperative myocardial infarction, repeat revascularization, 30-day readmission, and systematic graft patency, were not consistently collected. Finally, long-term survival and graft durability were not assessed.

## 6. Conclusions

This cohort demonstrates that a Heart Team-driven multivessel minimally invasive coronary revascularization program incorporating both complete surgical and planned hybrid strategies can be implemented with favorable observed perioperative outcomes in carefully selected patients. At the same time, the results should not be interpreted as evidence that MV-MIDCAB or HCR is superior to conventional CABG. Larger prospective comparative studies with standardized anatomical assessment, complete PCI documentation, systematic graft-patency assessment, and long-term clinical follow-up are required to determine the comparative effectiveness and durability of these strategies.

## Figures and Tables

**Figure 1:**
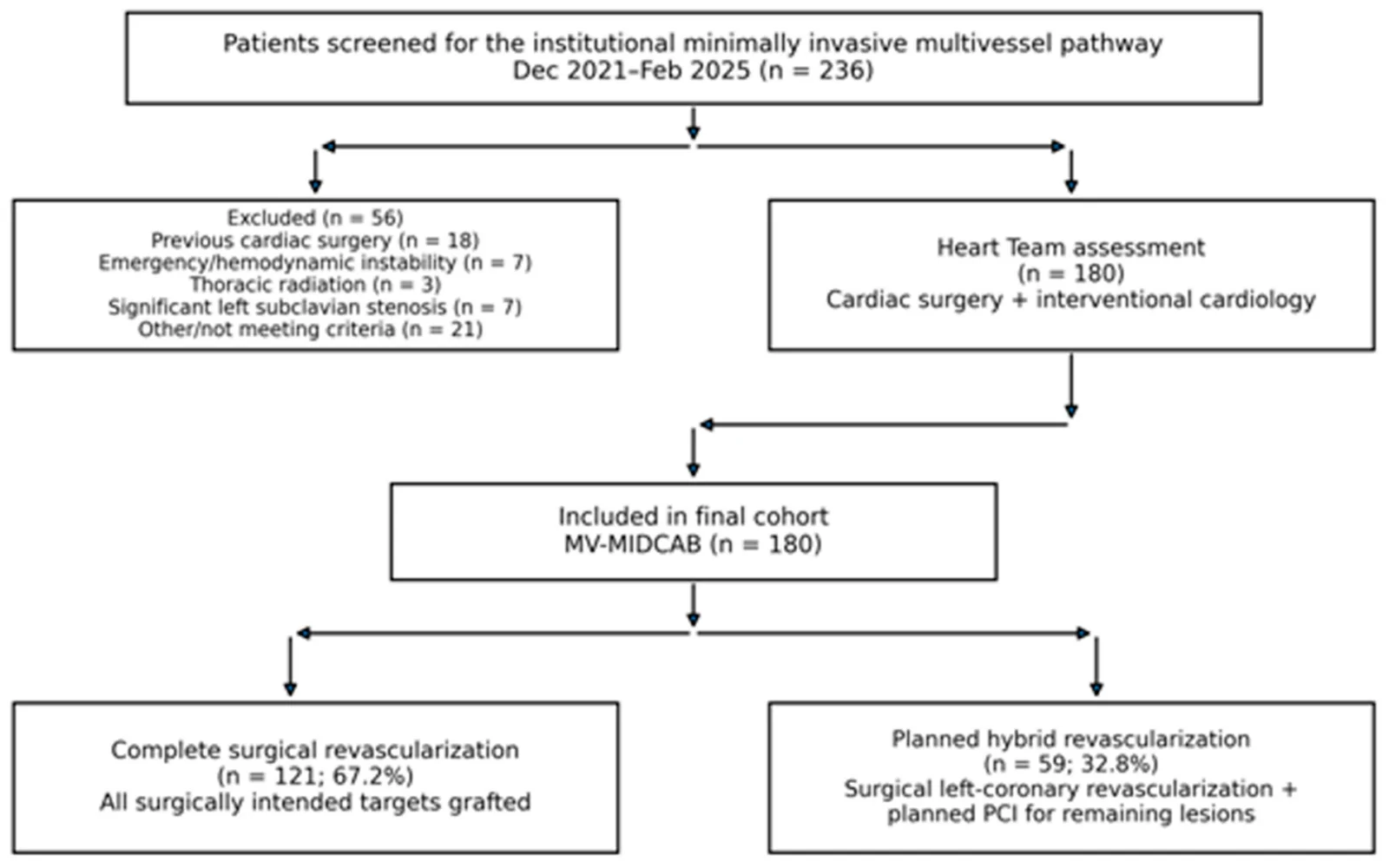
STROBE-aligned patient selection pathway [9].

**Figure 2:**
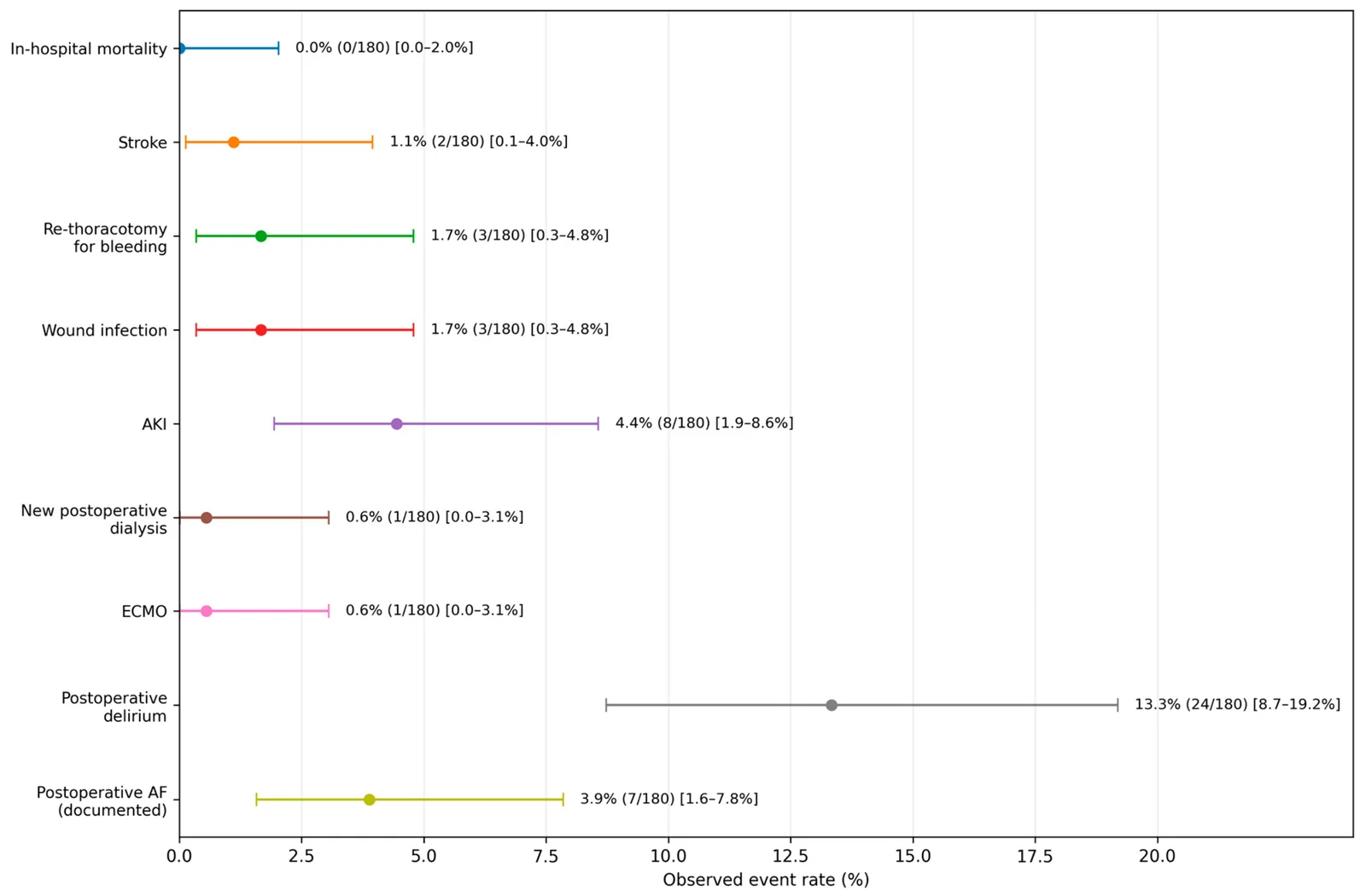
Postoperative results.

**Table 1:** Baseline clinical and anatomical characteristics.

Variable	Value	Available n
Age, years	68 (61–74)	179
BMI, kg/m^2^	27.0 [24.3–30.3]	179
Male sex	151/180 (83.9%)	180
EuroSCORE II, %	1.24 [0.86–1.67]	179
LVEF, %	55.0 [50.0–55.5]	179
LVEF > 55%	126/180 (70.0%)	180
Diabetes mellitus	52/180 (28.9%)	180
Hypertension	166/180 (92.2%)	180
Hyperlipidemia	159/180 (88.3%)	180
Previous stroke	14/180 (7.8%)	180
Preoperative atrial fibrillation	14/180 (7.8%)	180
Previous PCI	47/180 (26.1%)	180
Left main coronary disease	52/180 (28.9%)	180
NSTEMI	38/180 (21.1%)	180
STEMI	7/180 (3.9%)	180
HbA1c, %	5.6 [5.1–6.4]	173

Abbreviations: BMI, body mass index; EuroSCORE II, European System for Cardiac Operative Risk Evaluation II; LVEF, left ventricular ejection fraction; NSTEMI, non-ST-segment elevation myocardial infarction; PCI, percutaneous coronary intervention; STEMI, ST-segment elevation myocardial infarction.

**Table 2:** Operative strategy and conduit configuration.

Conduit/Strategy Audit	Available-Case Result
LIMA-only	30/179 (16.8%)
Bilateral IMA	42/180 (23.3%)
Total arterial revascularization	84/180 (46.7%)
T-graft configuration	151/180 (83.9%)
Thoracoscopic IMA harvest	83/180 (46.1%)

Abbreviations: IMA, internal mammary artery; LIMA, left internal mammary artery.

**Table 3:** Comparison of complete surgical and planned hybrid pathways.

Variable	Complete Surgical (n = 121)	Planned Hybrid (n = 58)
Age, years	68.0 [60.0–73.0]	68.5 [62.8–76.8]
Women	23/121 (19.0%)	6/58 (10.3%)
BMI, kg/m^2^	27.0 [23.7–30.5]	27.1 [25.4–30.0]
EuroSCORE II, %	1.32 [0.95–1.72]	1.10 [0.82–1.46]
LVEF, %	55.0 [50.0–55.0]	55.0 [55.0–60.0]
Diabetes mellitus	37/121 (30.6%)	14/58 (24.1%)
Previous PCI	28/121 (23.1%)	18/58 (31.0%)
Left main disease	40/121 (33.1%)	12/58 (20.7%)
NSTEMI	28/121 (23.1%)	10/58 (17.2%)
STEMI	5/121 (4.1%)	2/58 (3.4%)
Distal anastomoses, median [IQR]	3 (2–3)	2 (2–2)
Bilateral IMA	30/121 (24.8%)	12/58 (20.7%)
Total arterial revascularization	53/121 (43.8%)	31/58 (53.4%)
Thoracoscopic IMA harvest	54/120 (45.0%)	29/58 (50.0%)
Operation time, min	186 (169–200)	225 (181–274)
ICU stay, days	1 (1–2)	1 (1–1)
Hospital stay, days	4 (4–5)	4 (3–6)
Stroke	1/121 (0.8%)	1/58 (1.7%)
Re-thoracotomy for bleeding	2/121 (1.7%)	0/58 (0.0%)
AKI	2/121 (1.7%)	6/58 (10.3%)
Postoperative dialysis	1/120 (0.8%)	2/58 (3.4%)
Wound infection	2/121 (1.7%)	1/58 (1.7%)
Delirium	18/120 (15.0%)	6/58 (10.3%)
Postoperative AF documented	3/121 (2.5%)	4/58 (6.9%)
ECMO	1/121 (0.8%)	0/58 (0.0%)

Abbreviations: AF, atrial fibrillation; AKI, acute kidney injury; BMI, body mass index; ECMO, extracorporeal membrane oxygenation; IMA, internal mammary artery; ICU, intensive care unit; IQR, interquartile range; LVEF, left ventricular ejection fraction; PCI, percutaneous coronary intervention; STEMI/NSTEMI, ST-/non-ST-segment elevation myocardial infarction. One planned-hybrid patient had incomplete data for subgroup comparison and was therefore excluded from this table; subgroup denominators are based on available cases.

**Table 4:** Early postoperative outcomes.

Outcome	Observed Rate	Exact 95% CI
In-hospital mortality	0/180 (0.0%)	0.0–2.0%
Stroke	2/180 (1.1%)	0.1–4.0%
Re-thoracotomy for bleeding	3/180 (1.7%)	0.3–4.8%
Wound infection	3/180 (1.7%)	0.3–4.8%
AKI	8/180 (4.4%)	1.9–8.6%
New postoperative dialysis	1/180 (0.6%)	0.0–3.1%
Postoperative delirium	24/180 (13.3%)	8.7–19.2%
Postoperative AF documented	7/180 (3.9%)	1.6–7.8%
VA-ECMO	1/180 (0.6%)	0.0–3.1%
Conversion to CPB	1/180 (0.6%)	0.0–3.1%

Abbreviations: AF, atrial fibrillation; AKI, acute kidney injury; CPB, cardiopulmonary bypass; ECMO, extracorporeal membrane oxygenation; VA, venoarterial.

## Data Availability

The underlying patient-level dataset is not publicly available because it contains clinical information subject to institutional data-protection restrictions.
